# Crystal Structure and Molecular Mechanism of Phosphotransbutyrylase from *Clostridium acetobutylicum*

**DOI:** 10.4014/jmb.2109.09036

**Published:** 2021-09-25

**Authors:** Sangwoo Kim, Kyung-Jin Kim

**Affiliations:** 1 School of Life Sciences, BK21 FOUR KNU Creative BioSesearch Group, Kyungpook National University, Daegu 41566, Republic of Korea; 2 KNU Institute for Microorganisms, Kyungpook National University, Daegu 41566, Republic of Korea

**Keywords:** *Clostridium acetobutylicum*, phosphotransbutyrylase, butyryl-CoA, butyrate metabolism

## Abstract

Acetone-butanol-ethanol (ABE) fermentation by the anaerobic bacterium *Clostridium acetobutylicum* has been considered a promising process of industrial biofuel production. Phosphotransbutyrylase (phosphate butyryltransferase, PTB) plays a crucial role in butyrate metabolism by catalyzing the reversible conversion of butyryl-CoA into butyryl phosphate. Here, we report the crystal structure of PTB from the *Clostridial* host for ABE fermentation, *C. acetobutylicum*, (*Ca*PTB) at a 2.9 Å resolution. The overall structure of the *Ca*PTB monomer is quite similar to those of other acyltransferases, with some regional structural differences. The monomeric structure of *Ca*PTB consists of two distinct domains, the N- and C-terminal domains. The active site cleft was formed at the interface between the two domains. Interestingly, the crystal structure of *Ca*PTB contained eight molecules per asymmetric unit, forming an octamer, and the size-exclusion chromatography experiment also suggested that the enzyme exists as an octamer in solution. The structural analysis of *Ca*PTB identifies the substrate binding mode of the enzyme and comparisons with other acyltransferase structures lead us to speculate that the enzyme undergoes a conformational change upon binding of its substrate.

## Introduction

The acetone-butanol-ethanol (ABE) fermentation by *Clostridium acetobutylicum* undergoes either acid or solvent production depending on the cell growth phase [1]. During the initial growth phase (acidogenic phase), *C. acetobutylicum* produces acetate, butyrate, H_2_ and CO_2_, resulting in a pH decrease of culture medium [2-6]. However, in the stationary phase of growth, cell metabolism shifts to solvent production (solventogenic phase) [7-9]. During the solventogenic phase, ethanol and butanol are produced from acetyl-CoA and butyryl-CoA, with the formation of acetaldehyde and butyraldehyde in the presence of butyraldehyde dehydrogenase and then to butanol in the presence of butanol dehydrogenase. The metabolic pathway for formation of acids and solvents using acetyl-CoA involves three metabolites: acetyl-CoA, acetoacetyl-CoA and butyryl-CoA. During the acidogenic phase, the formation conversion of acetate and butyrate to acetyl-CoA and butyryl-CoA, respectively, occurs in a manner similar to both but requires different enzymes.

During the acidogenic phase in *C. acetobutylicum*, phosphotransbutyrylase (PTB, E.C. 2.3.1.8) and butyrate kinase (BUK, E.C. 2.7.2.7) play crucial roles in the production of butyrate from butyryl-CoA [10]. These enzymes also produce ATP by enzyme catalysis, and thus, they also play an important role in the energy metabolism of the organism. The shift from acid production to solvent production is accompanied by a decrease in activity of all the acid pathway enzymes and the induction of solvent pathway enzymes in *C. acetobutylicum* [1, 11]. The reassimilation of acids occurs after induction of solvent pathway enzymes. The CoA-transferase enzymes with broad specificity use either acetate or butyrate as CoA acceptor and convert butyrate and acetate to butyryl-CoA and acetyl-CoA, favoring solvent production.

**Fig. 1:**
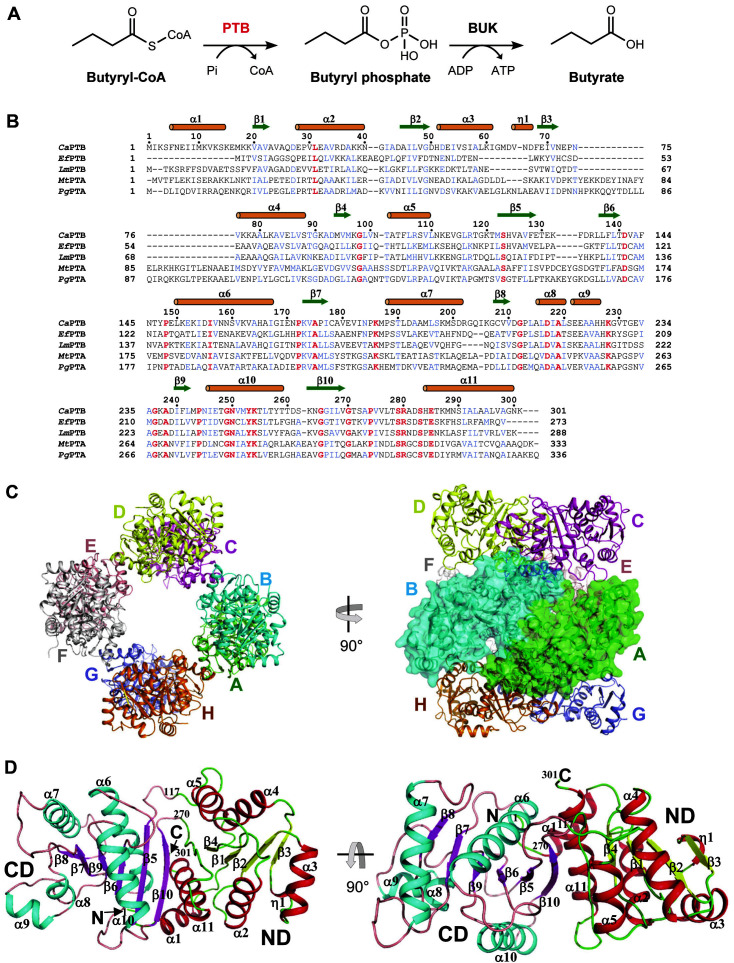
Overall structure of *Ca*PTB. (**A**) The catalytic reaction scheme diagram of butyrate biosynthesis. (**B**) Amino acid sequence alignment of PTBs. *Ca*PTB, *Lm*PTB, and *Ef*PTB represent PTB from *C. acetobutylicum*, *L. monocytogenes*, and *E. faecalis*, respectively, and *Mt*PTA and PpAPTA represent PTA from *M. thermophila* and *P. gingivalis*, respectively. The secondary structure elements are marked on top of the alignment based on the structure of *Ca*PTB. Conserved and highly similar residues are distinguished with the colors red and blue, respectively. (**C**) Octameric structure of *Ca*PTB. The *Ca*PTB structure is shown as a cartoon model. Each subunit is distinguished with the different colors of green, cyan, magenta, yellow, salmon, gray, light blue, and orange. (**D**) Monomeric structure of *Ca*PTB. The *Ca*PTB monomer is shown as a cartoon model. The N-terminal domain is shown with the colors red and yellow for α-helices and β-strands, respectively, and the C-terminal domain is shown with the colors cyan and magenta for α-helices and β-strands, respectively.

PTB catalyzes the conversion of butyryl-CoA through butyryl phosphate to butyrate in the butanoate pathway (Fig. 1A). In addition, the construction of synthetic pathways using *C. acetobutylicum* PTB and BUK as a metabolic engineering strategy to improve microbial biosynthesis of bioplastic polyhydroxyalkanoates (PHAs) and platform chemical adipic acid has been extensively studied [12-15]. It has also been reported that the engineered polythioester (PTE)-producing *E. coli* strains harboring the coding genes for butyrate kinase, phosphotransbutyrylase and PHA synthetase can produce various homopolymers consisting of different monomer units, such as 3-mercaptopropionate, 3-mercaptobutyrate, or 3-mercaptovalerate [16].

The structures of PTB and PTA had already been reported from several bacterial strains such as *Listeria monocytogenes*, *Enterococcus faecalis*, *Escherichia coli*, *Streptococcus pyogenes* [17], *Staphylococcus aureus*, *Bacillus subtilis* [18], and *Methanosarcina thermophila* [19, 20]. However, the structures of PTB and BUK from *C. acetobutylicum*, which is a well-known industrial host strain and biological producer of industrially important solvents, has not yet been reported. Therefore, we report the crystal structure of PTB from *C. acetobutylicum* (*Ca*PTB) to understand its molecular mechanism. Moreover, we reveal that the structure of *Ca*PTB has a unique oligomeric status. The substrate binding mode of *Ca*PTB and the conformational change upon binding of the substrate are also proposed.

## Materials and Methods

### Cloning, Expression, and Purification

The gene coding for phosphotransbutyrylase from *Clostridium acetobutylicum* (*Ca*PTB) was amplified using primers: forward, 5’- TATACATATGATTAAGAGTTTTAATGAAAT-3’ and reverse, 5’- GGTGCTCGAGTT ATTTATTGCCTGCAACTA-3’. The amplified DNA fragment was cloned into pET28a vector. The *Ca*PTB protein was expressed in the *E. coli* BL21(DE3)^T1R^ strain. The cells were cultured in an LB medium with kanamycin at 37°C. At OD_600_ = 0.7 at 600 nm, the cells were induced by adding 0.1 mM isopropyl-1-thio-β-D-galactopyranoside (IPTG) and further cultured for 20 h at 18°C. The harvested cell pellet was resuspended in buffer A (40 mM Tris-HCl, pH 8.0, 5 mM β-mercaptoethanol) and the cell lysis was accompanied by ultrasonication. The cell lysate was centrifuged at 12,000 ×*g* for 20 min and the cell debris was removed. The protein was purified using Ni-NTA agarose. To remove trace amounts of contaminants, size exclusion chromatography (Superdex200, GE Healthcare, USA) was applied. The purified protein was concentrated to 40 mg/ml for crystallization.

### Crystallization, Data Collection, and Structure Determination

Initial crystallization screening was performed using commercial crystal screening kits such as Index, PEG/Ion, Crystal Screen (Hampton Research, USA), and Wizard I & II (Rigaku, Japan) and by employing the hanging-drop vapor-diffusion method at 20°C. Each drop was prepared by mixing 1.0 μl protein solution with 1.0 μl reservoir solution and then equilibrating against 50 μl of the reservoir solution. The *Ca*PTB crystals were obtained from crystallization condition of 20% PEG3350, 0.2 M lithium sulfate, and 0.1 M HEPES (pH 7.5). For cryo-protection, 30% glycerol was added to the crystallization solution. The data were collected at 100 K at the 7A beamline of the Pohang Accelerator Laboratory (Korea), and the collected data were processed using the HKL2000 suite [21]. The *Ca*PTB crystal belonged to monoclinic space group *P*2_1_, with unit cell parameters of *a*=94.7, *b*=143.4 Å, *c*=113.3 Å, β=94°. Assuming one molecule of *Ca*PTB per asymmetric unit, the crystal volume per unit of protein mass was approximately 2.97 Å3 Da^-1^, which corresponds to a solvent content of approximately 58.7% [22].

The crystal structure of *Ca*PTB was solved by molecular replacement using MOLREP [23]. The structure of phosphotransacylase from *E. faecalis* (PDB code 1YCO, 42% sequence identity) was used as a search model. The final model building was performed using the program WinCoot [24] and the refinement was performed with REFMAC5 [25]. The geometric parameters of the final model were validated by using PROCHECK [26] and MolProbity [27]. The data statistics are summarized in Table 1. The refined model of *Ca*PTB was deposited in the Protein Data Bank (PDB code 7VG9).

### Size-Exclusion Chromatography

Analytical size-exclusion chromatography was performed using the Superdex 200 Increase 10/300 GL column (GE Healthcare Life Sciences). The column was equilibrated with a buffer containing 40 mM Tris–HCl, pH 8.0, and 150 mM NaCl. A protein sample of 0.5 ml with 1 mg/ml concentration was used for analysis. Standard samples, such as ferritin (440 kDa), conalbumin (75 kDa), carbonic anhydrate (29 kDa), and ribonuclease A (13.7 kDa), were used for calculation of the molecular weight.

## Results and Discussion

### Overall Structure of *Ca*PTB

To understand the molecular mechanism of the butyrate biosynthesis of *C. acetobutylicum*, we determined the crystal structure of phosphotransbutyrylase from *C. acetobutylicum* (*Ca*PTB, phosphate butyryltransferase) at a 2.9 Å resolution (Figs. 1B, 1C). It is assumed that the low resolution of the *Ca*PTB structure is caused by the high solvent content percentage and the unique oligomeric state. The asymmetric unit contains eight *Ca*PTB molecules forming an octamer (Fig. 1C). The *Ca*PTB monomer consists of two α/β domains, which can be divided into the N-terminal domain (ND, residue Met1–Arg117 and Ser272–Lys301) and the C-terminal domain (CD, residue Thr118–Thr271) (Figs. 1B, 1D). These domains show a side-by-side arrangement and form a continuous β-sheet-like shape surrounded by α-helices. An active site cleft was formed between two domains that is known to show an open-close conformation change during substrate-binding [28]. The N-terminal domain consists of a four-stranded parallel β-sheet (β1–β4) with six α-helices (α1–α5 and α11) and one 3_10_-helix (η1). The C-terminal domain is composed of a four-stranded parallel β-sheet (β7–β10) with one β-hairpin (β5–β6) and five helices (α6–α10).

**Fig. 2:**
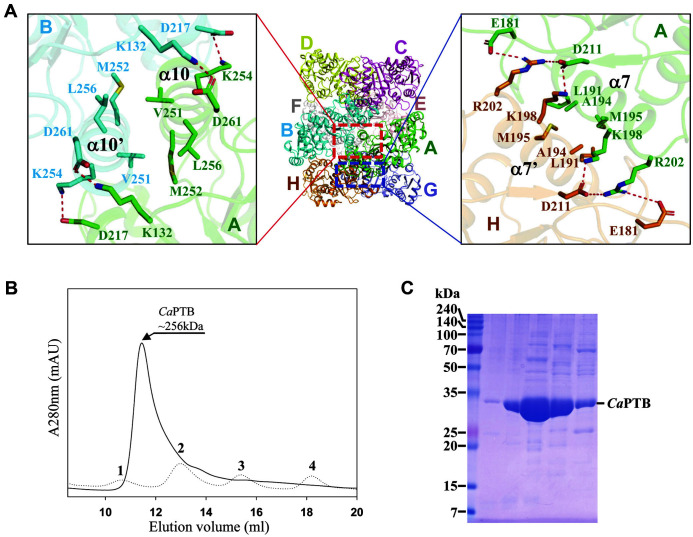
Oligomeric status of *Ca*PTB. (**A**) Interfaces for octamer formation of *Ca*PTB. The residues for hydrogen bonding are presented with a stick model. The hydrogen bonds are shown as red-colored dotted lines. (**B**) Size-exclusion chromatography analysis of *Ca*PTB. 1, 2, 3 and 4 indicate standard samples of ferritin (440 kDa), aldolase (158 kDa), ovalbumin (44 kDa), and ribonuclease A (13.7 kDa), respectively. (**C**) SDS-PAGE of purified *Ca*PTB. The *Ca*PTB monomer with a molecular weight of approximately 32 kDa is indicated.

The *Ca*PTB dimer is formed by hydrogen bonds and salt bridge of the loop of β13– β14 and helix α8 and α10 between C-terminal domains in subunits A–B, including Lys132–Asp261 and Asp217–Lys254 (Fig. 2A). Furthermore, the dimer from the subunits A–B, C–D, E–F and G–H interact through helix-to-helix hydrophobic interactions using the hydrophobic residues including Val251, Met252 and Leu256. PISA software calculated the surface area at 6029 Å^2^ [29].

The asymmetric unit of our *Ca*PTB crystal contained an octameric structure (Figs. 1B, 2A). When the crystal structure was solved, we considered that the asymmetric unit of the *Ca*PTB crystal contained four dimers, because the PTB enzymes are known to function as a dimer. However, previous studies suggested that *Ca*PTB can form an octamer [30, 31], and the size-exclusion chromatography experiment confirmed that *Ca*PTB exists as an octamer in solution (Figs. 2B, 2C). The octamer structure of the *Ca*PTB consists of four dimers that are connected in a caterpillar track-like arrangement. The contacts for octamerization are mediated by hydrogen bonds and salt bridges between residues including Glu181–Arg202, Lys198–Asp211 and Asp211–Arg202. The hydrophobic interactions also heavily contribute to the octamerization using residues including Leu191, Ala194 and Met195, and these residues are located mainly in α7 of the C-terminal domains in each subunit (Fig. 2A). The octamer interface between each subunit (A–H, B–C, D–E, and F–G) buries a solvent-accessible area of 679, 721, 708, and 730 Å^2^ as calculated by PISA [29]. The average solvation free energy gain (Δ^i^G) for the octamer formation of the interface was -7.9 kcal/mol with an average *p*-value of 0.165. This interface received a complex formation significance score (CSS) of 0.087. The results indicate that the interface plays an essential role in complex formation and the dimeric interface with specific interactions is not an artefact of crystal packing.

### Active Site of *Ca*PTB

**Fig. 3:**
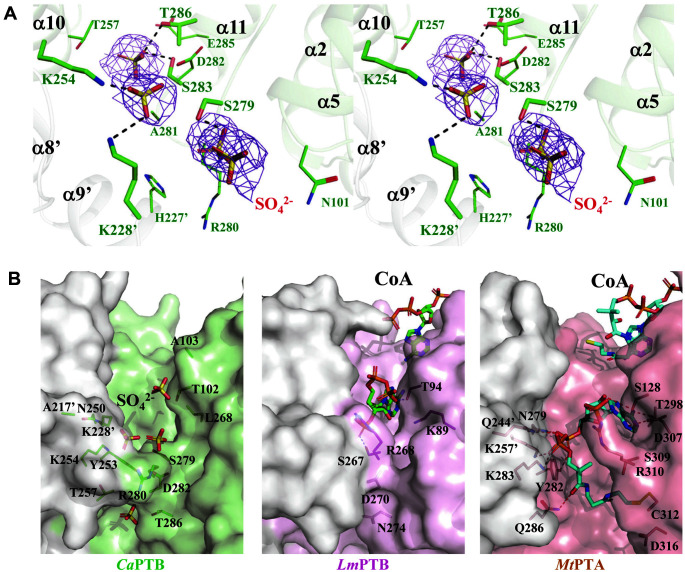
Active site of *Ca*PTB. (**A**) The active site of *Ca*PTB. Four sulfate ions are shown with a stick model in orange. The Fo−Fc electron density map is shown as a purple mesh and is contoured at 3.5 σ. The residues for hydrogen bonding and van der Waals contact are presented with stick and line models, respectively. The hydrogen bonds are shown with black-colored dotted lines. (**B**) The active sites cleft of *Ca*PTB, *Lm*PTB, and *Mt*PTA. CoA binding residues for hydrogen bond formation in *Lm*PTB and *Mt*PTA are presented as a line model with the colors magenta and cyan, respectively, and their corresponding residues in *Ca*PTB are shown with in green. Sulfate ions and CoA molecules are represented as orange, green, and cyan-colored stick models, respectively. The hydrogen bonds are shown as red-colored dotted lines.

To understand the reaction mechanism and substrate binding mode of *Ca*PTB, we first attempted to determine the complex structure with its butyryl-CoA substrate. However, neither co-crystallization nor soaking with the substrate were successful. Instead, we observed four sulfate ions, which were added during the crystallization procedure, at the active site cleft formed between the two domains (Fig. 3A). The sulfate ions were stabilized by hydrogen bonds with the residues Lys254, Ser279, Ser283, Thr286, and Lys228. When we compared the structure of *Ca*PTB with that of PTB from *L. monocytogenes* (*Lm*PTB, PDB code 3U9E, 39% amino acid identity, 1.8 Å RMSD) and PTA from *M. thermophila* (*Mt*PTA, PDB code 2AF4, 19% amino acid identity, 4.3 Å RMSD) [19, 20], the pyrophosphate-moiety of the bound CoA in *Lm*PTB and *Mt*PTA were located in the position similar to the sulfate ions bound in *Ca*PTB (Fig. 3B). Two structures of phosphotransacetylase, *Lm*PTB and *Mt*PTA, showed one CoA bound to the proposed active site cleft and an additional CoA bound to the periphery of the cleft. In these structures, the CoA bound to the cleft between the two domains is mediated by a series of hydrogen bonds and extensive van der Waals interactions, while other CoA bound around the cleft have relatively weaker interactions. In the active site of *Mt*PTA, CoA were stabilized by the residues Ser128, Asn279, Tyr282, Lys283, Gln286, Thr298, and Cys312 from subunit A, and the residues Gln244 and Lys257 from subunit B. According to the catalytic mechanism proposed in *Mt*PTA, Asp316 catalyzes a sulfhydryl proton in CoA, whereby the thiolate anion attacks the carbonyl carbon of acetyl phosphate. Furthermore, the substrate is stabilized by Ser309, and the generated phosphate ions return Asp316 to a deprotonated state for the next round of catalysis. The Asp316 residue, which attacks the substrate in PTA, is present as Thr286 in *Ca*PTB, and nearby Asp282 is a conserved residue in PTB and is expected to act on the substrate (Figs. 1B, 3B). These observations indicate that *Ca*PTB, *Lm*PTB, and *Mt*PTA might accommodate similar substrates with somewhat different modes.

### Comparison of Similar Acyltransferases Proteins

We then attempted to compare the *Ca*PTB structure with its structural homologues among phosphate acetyl/butyryltransferases, and the Dali server search showed that *Ca*PTB was highly structurally similar to members of acyltransferases such as phosphotransbutyrylase from *L. monocytogenes* (*Lm*PTB, PDB code 3U9E) and *E. faecalis* (*Ef*PTB, PDB code 1YCO) and phosphotransacetylase (PTA, phosphate acetyltransferase) from *M. thermophila* (*Mt*PTA, PDB code 2AF3) [19, 20] and *P. gingivalis* (PgPTA, PDB code 6IOX) [32] with RMSD for related elements ranging from 1.8 to 4.3 Å and 19-39% amino acid identity (Fig. 1B).

**Fig. 4:**
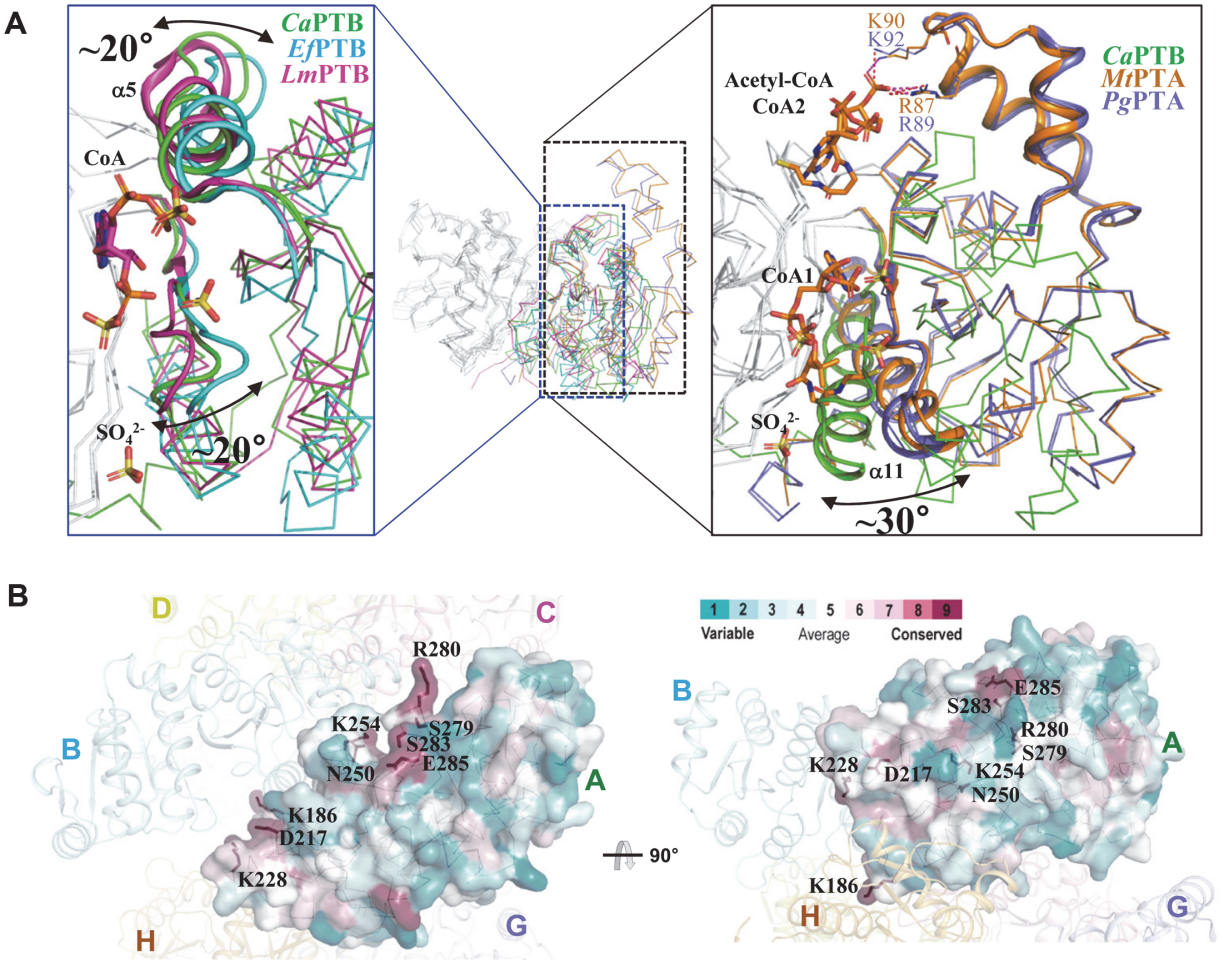
Structural comparison of *Ca*PTB with other PTBs and PTAs. (**A**) Superimposition of *Ca*PTB with other structural homologues. *Ca*PTB, *Ef*PTB, *Lm*PTB, *Mt*PTA, and PgPTA structures are presented with a ribbon diagram and distinguished with different colors. The regions with structural differences are highlighted with a cartoon diagram. (**B**) Surface conservation mapping of *Ca*PTB. The *Ca*PTB structure was shown with a surface conservation model. Highly conserved residues are shown as a stick model and labeled.

The superposition of the *Ca*PTB structure with those of structurally similar enzymes revealed that the C-terminal domain of *Ca*PTB was well superposed with other enzymes. However, α5 and α11 of the N-terminal domain of *Ca*PTB was rotated by about 20 to 30 degrees from the other enzymes. Because the structure of *Ca*PTB is an apo form, whereas other compared structures are complexed form with the substrate, the structural difference at the α5 and α11 might indicate that PTB undergoes an open-close conformational change upon the substrate binding (Fig. 4A). Moreover, we observed a local structural difference at the lid region, where *Ca*PTB has an additional 20 residues compared with other enzymes. These additional residues form a helix-turn-helix structure. These helices of PTA show more of an open conformation compared with the corresponding helices of PTB, which seems to be caused by a structural change due to substrate specificity along with the lid region. A mapping of the conserved residues on the surface of *Ca*PTB was performed with multisequence alignment of PTBs and PTAs using ClustalW [33] and ConSurf [34]. The conserved residues were located at the residues Lys186, Asp217, Lys228, Asn250, Lys254, Ser279, Arg280, Ser283, and Glu285 (Fig. 4B). While the residues Asp217 and Lys254 contributed to the dimerization, other residues were involved in the enzyme catalysis and the substrate binding. The Lys254 residue was involved in both dimerization and substrate binding. Interestingly, residues involved in the octamer formation, such as Asp181, Arg202, and Asp211, were not conserved, which might be due to the fact that *Ca*PTB possesses it own unique octameric oligomeric status.

In summary, we report the crystal structure of *Ca*PTB, a crucial enzyme involved in butyrate biosynthesis. Unlike other PTBs, *Ca*PTB forms an octamer, which is mediated by the tetramerization of four dimers. By comparing it with other structural homologues, we elucidated that *Ca*PTB stabilizes its substrate using conserved residues and undergoes a conformational change upon substrate binding. Finally, the conservation mapping analysis of PTB shows that, although residues involved in the enzyme catalysis and substrate binding are highly conserved among PTB enzymes, *Ca*PTB has quite unique residues for octamerization, which is consistent with the fact that *Ca*PTB forms a unique octameric oligomer.

## Figures and Tables

**Table 1:** Data collection, phasing and refinement statistics.

	*Ca*PTB
PDB code	
Data collection	
Space group	*P*2_1_
Cell dimensions	
*a, b, c* (Å)	94.7, 143.4, 113.3
α, β, γ (°)	90.0, 94.0, 90.0
Resolution (Å)	50.00-2.90 (2.95-2.90)
*R*_sym_ or *R*_merge_	33.5 (11.5)
I / *σΙ*	18.6 (4.4)
Completeness (%)	95.9 (90.7)
Redundancy	3.0 (2.3)
Refinement	
Resolution (Å)	50.00-2.90
No. of reflections	59813
*R*_work_ / *R*_free_	17.2 / 23.8
No. of atoms	18443
Protein	18035
Sulfur ion	240
Water	158
*B*-factors	37.9
Protein	40.6
Sulfur ion	77.1
Water	28.3
R.m.s. deviations	
Bond lengths (Å)	0.013
Bond angles (°)	1.664
Ramachandran plot	
Most favored (%)	98.2
Additional allowed (%)	1.8

*Values in parentheses are for highest-resolution shell.
